# Editorial: Immune system mechanisms impacting the onset of epilepsy and spontaneous seizures

**DOI:** 10.3389/fnmol.2024.1487871

**Published:** 2024-09-10

**Authors:** Claudia Espinosa-Garcia, Erfan Bahramnejad, Yi Li

**Affiliations:** ^1^Department of Neurology, Yale University, New Haven, CT, United States; ^2^Department of Pharmacology, University of Arizona, Tucson, AZ, United States; ^3^Department of Neurology and Neurological Sciences, Stanford University, Palo Alto, CA, United States

**Keywords:** epilepsy, immune system, epileptogenesis, neuroinflammation, microglia, infiltrating monocytes, complement C3

## 1 Introduction

Epilepsy, a chronic neurological condition affecting more than 50 million people worldwide (in the U.S. alone almost three million Americans), is characterized by spontaneous recurrent seizures and associated with cognitive decline and behavioral comorbidities (WHO, 2024; CDC, 2024). Despite the major advances made in therapeutics, more than 30% of epilepsy patients suffer from poor control of seizures throughout life (Kalilani et al., 2018). Most of existing drugs are designed to treat the symptoms, but do not prevent epilepsy in people at risk nor modify the disease progression (Galanopoulou et al., 2021; Chen et al., 2018). The development of epilepsy, or epileptogenesis, is a gradual process (Pitkänen et al., 2015); therefore, a better understanding of the underlying pathological processes might lead to identification of more effective targeted therapies or novel avenues to reduce or prevent seizures. Experimental and human evidence builds a solid foundation of a direct link between epileptogenesis and inflammation (Vezzani et al., 2023; Dingledine et al., 2024). This Research Topic provides new insights into the inflammatory and immune mechanisms that might contribute to the onset of epilepsy and spontaneous seizure occurrence.

## 2 Role of complement C3 in epileptogenesis

The activation of the complement system has been reported to occur in experimental models of status epilepticus (SE) and human temporal lobe epilepsy (TLE). In previous studies complement C3 levels persisted elevated after SE or at the chronic stage of TLE, correlating with seizure severity and cognitive deficits (Aronica et al., 2007; Schartz et al., 2018; Kharatishvili et al., 2014). Here, Schartz et al. demonstrate that C3 knockout mice subjected to pilocarpine-induced SE did not display memory deficits nor astrogliosis, suggesting that C3 ablation prevent cognitive decline during epileptogenesis. These findings nominate complement C3 as a disease-enhancing molecule that might contribute to the development of epilepsy, and hence a novel therapeutic target for epileptogenesis prevention.

## 3 Neuroinflammation in epileptogenesis

Clinical and experimental lines of evidence support a crucial role for neuroinflammation in the development of epilepsy. For instance, Li et al. reviewed relevant pro-inflammatory mediators and inflammatory pathways that might lead to epileptogenesis, ranging from microglia and astrocytic activation, brain blood barrier dysfunction, and systemic inflammatory events (e.g., monocyte infiltration to the brain). Furthermore, Bröer and Pauletti summarized the beneficial and harmful role of resident microglia and infiltrating monocytes, which upon activation, influence seizure initiation and disease progression. Together this evidence strongly emphasize that inflammatory factors and microglia/infiltrating monocytes could be used as potential biomarkers to identify patients at risk or targets for therapeutic approaches in the treatment of epilepsy.

## 4 A Src tyrosine kinase inhibitor as a novel avenue for the treatment of epilepsy

Anti-inflammatory drugs targeting neuroinflammation show promising results for disease modification (Vezzani et al., 2024). Recent reports implicate Src tyrosine kinases in epilepsy-related neuroinflammation (Liu et al., 2022; Sharma et al., 2021). In this study, Rao et al. examine the protective effects of inhibiting Src via saracatinib in the rat kainic acid model of SE. Treatment with saracatinib mitigated microgliosis and reactive astrocytes, prevented neurodegeneration, and reduced cortical glial scar. Given its efficacy in targeting epileptogenic processes, saracatinib could be a promising disease-modifying agent to prevent the development and progression of epilepsy.

## 5 Using bioinformatics to identify new hub genes linked to epilepsy

Neuronal cell death–apoptosis, necroptosis, pyroptosis, ferroptosis, or autophagy–can worsen seizure occurrence and epilepsy progression. Pharmacological inhibition of neuronal cell death has proven to be an effective therapy for SE (Du et al., 2022). Via a differential expression analysis, Wang et al. identified five apoptosis-related genes CD38, FAIM2, IL1B, PAWR, and S100A8 as potential diagnostic biomarkers. Despite small sample size in the data sets, the constructed diagnostic model indicated a remarkable accuracy in patients with TLE compared to controls. Future experimental studies are needed to validate these bioinformatic findings.

## 6 Early-life environmental insults prime epileptogenesis

Neurofibromatosis type 1 (NF1) patients have an increased risk to develop epilepsy in adulthood (Hébert et al., 2024). Early-life environmental insults, including cerebral ischemia, brain trauma, or infection, share common pathological pathways involving innate immune activation and neuroinflammation (Semple et al., 2020). To address the relevance of early immune activation in epilepsy development, Faidi and Reid used lipopolysaccharide to prime the brain for later spontaneous seizures and cognitive deficits in a mouse model of NF1. Their results showed that early immune activation promotes seizure susceptibility, without effects on learning/memory, suggesting early-life environmental insults are an important risk factor for NF1-associated epilepsy.

## 7 New onset refractory status epilepticus and febrile infection-related epilepsy syndrome

By consensus, new onset refractory status epilepticus (NORSE) is defined as “a clinical presentation characterized by new onset of refractory SE, in a patient without active epilepsy or other preexisting relevant neurological disorder, and without a clear acute or active structural, toxic or metabolic cause”; in contrast, febrile infection-related epilepsy syndrome (FIRES) is a subcategory of NORSE with a preceding febrile infection (Hirsch et al., 2018). In both conditions, survivors have a poor response to antiepileptic medications leading to a high seizure burden and poor quality of life (Wickstrom et al., 2022). In this review, Champsas et al. analyzed the supporting clinical, preclinical, emerging treatments (e.g., anesthetics, immunological and dietary approaches) and outcome data that highlight the understudied role of immune-mediated inflammatory process in the NORSE/FIRES pathophysiology. Importantly, authors propose future directions for *in vivo* and *in vitro* epilepsy research and provide a call-to-action for experts in Neurology, Neuroscience, Immunology, and other Biomedical Sciences to work together for improving patients outcomes.

The molecular and cellular mechanisms underlying epileptogenesis include but are not restricted to neuroinflammation. This Research Topic examined important immune players, risk factors and interventions for neuroinflammation in the context of epilepsy development. With further investigation of inflammatory and immune pathways, the field will get closer to achieving the ultimate goal of finding useful disease-modifying agents for patients with epilepsy.
